# Analysis of spontaneous reporting of suspected adverse drug reactions for non-analgesic over-the-counter drugs from 2008 to 2017

**DOI:** 10.1186/s40360-019-0338-2

**Published:** 2019-10-18

**Authors:** Josipa Bukic, Doris Rusic, Petar Mas, Deni Karabatic, Josko Bozic, Ana Seselja Perisin, Dario Leskur, Darko Krnic, Sinisa Tomic, Darko Modun

**Affiliations:** 1 Department of Pharmacy, University of Split School of Medicine, Soltanska 2, 21000 Split, Croatia; 2 Agency for Medicinal Products and Medical Devices of Croatia, Ksaverska cesta 4, 10 000 Zagreb, Croatia; 3 Department of Pathophysiology, University of Split School of Medicine, Soltanska 2, 21000 Split, Croatia

**Keywords:** OTC drugs, Pharmacovigilance, Adverse drug reactions, Drug safety, Spontaneous reporting

## Abstract

**Background:**

Adverse drug reaction (ADR) reporting practices by health care professionals remain poor. Over-the-counter (OTC) drugs are perceived as safe; however, they can also cause ADRs. The objective of this study was to analyze ADR reporting for OTC drugs in a 10-year period, in order to evaluate frequency of ADRs, population that ADRs most affect and reporters of ADRs of OTC drugs in Croatia.

**Methods:**

Spontaneously reported ADRs of non-analgesic OTC drugs, collected from January 2008 to December 2017 were analyzed. Data was obtained from Agency for Medicinal Products and Medical Devices of Croatia (HALMED).

**Results:**

There were 547 ADRs of OTC drugs reported in total and an increase in number of reports through the years was observed. Pharmacists reported 45.4% of all ADRs, and were most frequent reporters (*p* < 0.001). In 2017 majority of reports, 62 (49.2%), were obtained from consumers. ADRs were most frequently observed in patients aged 70 years and older (15% of ADRs). Five percent of all reports were accidental exposures among children.

**Conclusions:**

Pharmacists most frequently reported ADRs of OTC drugs and consumers’ awareness of ADR reporting has risen. Other health care professionals (e.g., nurses and dentists) must be offered proper education in order to improve reporting practice of ADRs. Health care professionals should address concerns about OTC drug safety in elderly and children.

## Background

The World Health Organization (WHO) defines self-medication as “the selection and use of medicines by individuals to treat self-recognized illnesses or symptoms” [1]. There are many benefits of self-medication, for both patients and health professionals, among which include physicians avoiding unnecessary consultations, increased advisory role of pharmacists, and patients becoming responsible for their own health. However, risks of polypharmacy (especially in the elderly), inappropriate use of OTC drugs and masking of symptoms of other diseases can also be attributed to self-medication [2, 3]. Furthermore, OTC drugs, while perceived safe, can also cause adverse drug reactions (ADRs) and the public should become aware that the safety of OTC drugs should be treated similarly as the safety of prescribed drugs [4].

ADRs influence consumers’ quality of life, whereby ADRs may increase health care costs and are a well-recognized reason for hospitalization and cause of mortality [5, 6]. In order to improve knowledge about drug safety, spontaneous reporting of suspected ADRs has been encouraged. The spontaneous reporting of ADRs has been one of the most essential methods for monitoring the safety of marketed drugs, and remains one of the most efficient methods to detect serious or new ADRs [7, 8]. However, we are still witnessing under-reporting of ADRs, even those characterized as serious [7, 9–11].

Several studies investigated attitudes towards ADR reporting and the ADR reporting practice of health care professionals [12, 13]. Namely, positive attitudes about ADR reporting were observed, but reporting practice was poor [14]. Some of the reasons listed by pharmacists and physicians for not reporting ADRs in their daily practice were the perception that a particular ADR was too trivial or too well known and the opinion that serious ADRs were already documented [15, 16].

Therefore, the aim of this study was to analyze ADR reporting for non-analgesic OTC drugs in a 10-year period to evaluate the frequency of ADRs, the population that ADRs most affect and reporters of ADRs of non-analgesic OTC drugs in Croatia.

## Materials and methods

### Study drugs

There were 469 OTC drugs with different trade names marketed in Croatia in November 2018, among which are 150 analgesics and 319 other OTC drugs. ADR reports of analgesics were excluded from this study due to the fact that data on ADRs for analgesics were previously published [17]. We collected and analyzed reports from January 2008 to December 2017 for the current study.

### Data source

Spontaneously reported ADR data were obtained from the Agency for Medicinal Products and Medical Devices of Croatia (HALMED). The provided data were extracted from VigiBase, the WHO global database of individual case safety reports (ICSRs). Each report contained ADRs coded according to the Medical dictionary for Regulatory Activities (MedDRA) terminology. MedDRA version 21.0 was used for this study. In order to exclude ADR reports on prescription drugs, we searched the database using the trade names of OTC drugs.

### Data analysis

The following data were analyzed: report year, reporter qualification (pharmacist, physician, consumer/non health professional, or other health professional), patient gender and age, ADR seriousness (including non-serious, caused/prolonged hospitalization, life threatening, as well as other and death), active substance name, reported reaction (MedDRA), concomitant therapy, and the Anatomical Therapeutic Chemical (ATC) classification of the drugs. The first level of the anatomical main group was used for this study. The proportion of ADRs for included drugs relative to all ADR reports in each year were calculated. The data on the total number of reports were obtained from HALMED’s annual reports of spontaneous reporting of ADRs.

The aforementioned data was exported into a Microsoft Excel (Microsoft Office, 2016) spreadsheet. We reported descriptive statistics as whole numbers and proportions where appropriate. Statistical analysis was performed using MedCalc software for Windows (v.11.5.1.0, MedCalc Software, Ostend, Belgium). The Chi-square test was used for comparing categorical variables. Statistical significance was set at *p* < 0.05.

## Results

**Fig. 1:**
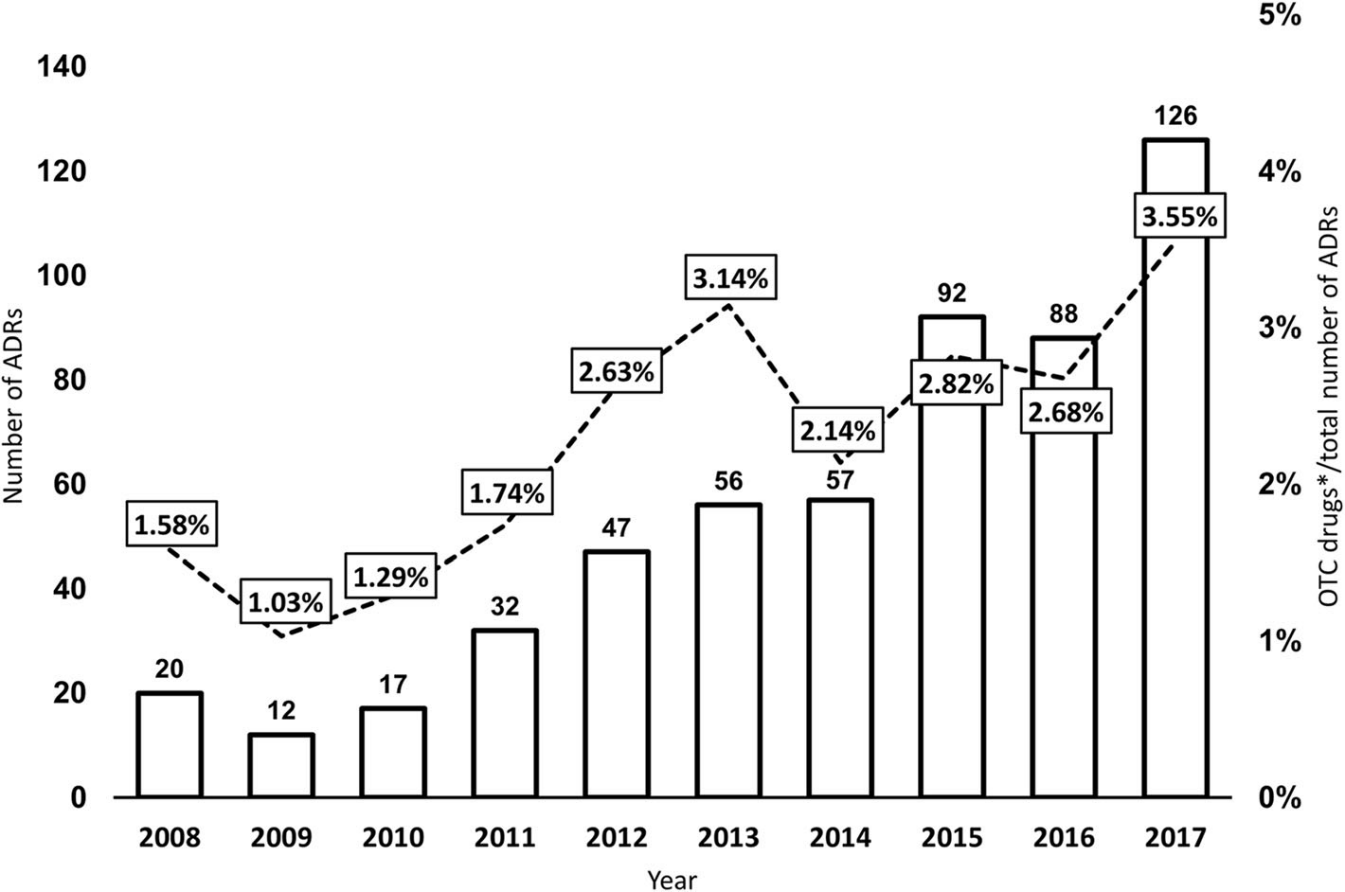
Number and proportion of ADR reports of OTC drugs

There were 547 ADR reports of OTC drugs reported to HALMED in total, during the examined period. The number of ADR reports of OTC drugs and percentages of the total number of ADRs of OTC drugs of spontaneously reported ADRs in Croatia are presented in Fig. 1. An increase in the number of reports through 2013–2017 was observed in that almost 80% of ADRs of OTC drugs were reported during that time.

Furthermore, 319 included trade name drugs accounted for 106 different active substances. For 61.3% of these active substances, there was at least one ADR report in the HALMED database. The list of OTC drugs along with their number of ADR reports is available as Additional file 1.

The main characteristics of ADR reports are shown in Table 1. Pharmacists reported ADRs of OTC drugs most frequently (*p* < 0.001), physicians and consumers had equally contributed, and other health care professionals contributed to a minor number of reports. In 2017, the majority of reports for OTC drugs, 62 (49.2%), were obtained from consumers. This number accounted for 44.9% of all reports obtained from consumers in the studied period. In general, there were more ADRs reported for female patients than male patients. ADRs were most frequently observed in patients aged 70 years and older. However, a large number of ADRs were observed among children under 10 years of age and 28 of those ADR reports (5% of all reports) were accidental exposure to product by child. This was mainly due to a collaboration beginning in 2016 between HALMED and the Poison Control Centre regarding the collection of drug overdose information. In terms of seriousness criteria, 97 (17.7%) ADR reports were classified as serious and the majority of reports included a non-serious ADR.

**Table 1:** Main characteristics of ADR reports of OTC drugs to HALMED during a 10-year period

Characteristic	*N* (%)	*p* value*
Reporter qualification		< 0.001
Pharmacist	248 (45.4)	
Physician	149 (27.2)	
Consumer/non health professional	138 (25.2)	
Other health professional	10 (1.8)	
Missing	2 (0.4)	
Patient gender		< 0.001
Male	178 (32.6)	
Female	358 (65.4)	
Missing	11 (2.0)	
Patient age (years)		< 0.001
< 10	72 (13.2)	
10–19	19 (3.5)	
20–29	55 (10.1)	
30–39	77 (14.1)	
40–49	46 (8.4)	
50–59	63 (11.5)	
60–69	65 (11.9)	
≥ 70	82 (15.0)	
Missing	68 (12.3)	
Non-serious ADRs	450 (82.3)	< 0.001
Serious ADRs	97 (17.7)	
Caused/prolonged hospitalization	16 (16.6)	
Life threatening	8 (8.2)	
Other	72 (74.2)	
Death	1 (1.0)	

*Chi square test

ADR report analysis according to MedDRA System Organ Class (SOC) showed that the most frequently reported ADRs were gastrointestinal (*N* = 154; 23.4%), skin and subcutaneous tissue (*N* = 145; 22.0%) and nervous system disorders (*N* = 104; 15.8%). Overall, 36.5% of ADR reports were for drugs classified in ATC group R (Respiratory system), 18.3% were from group D (Dermatologicals) and 17.5% from group A (Alimentary tract and metabolism). Other reports, in minor proportions, included drugs from group G (Genitourinary system and sex hormones) with 14.4%, group C (Cardiovascular system) with 8.3%, group N (Nervous system) with 4.1% and less than 1% reports from group S (Sensory organs).

**Fig. 2:**
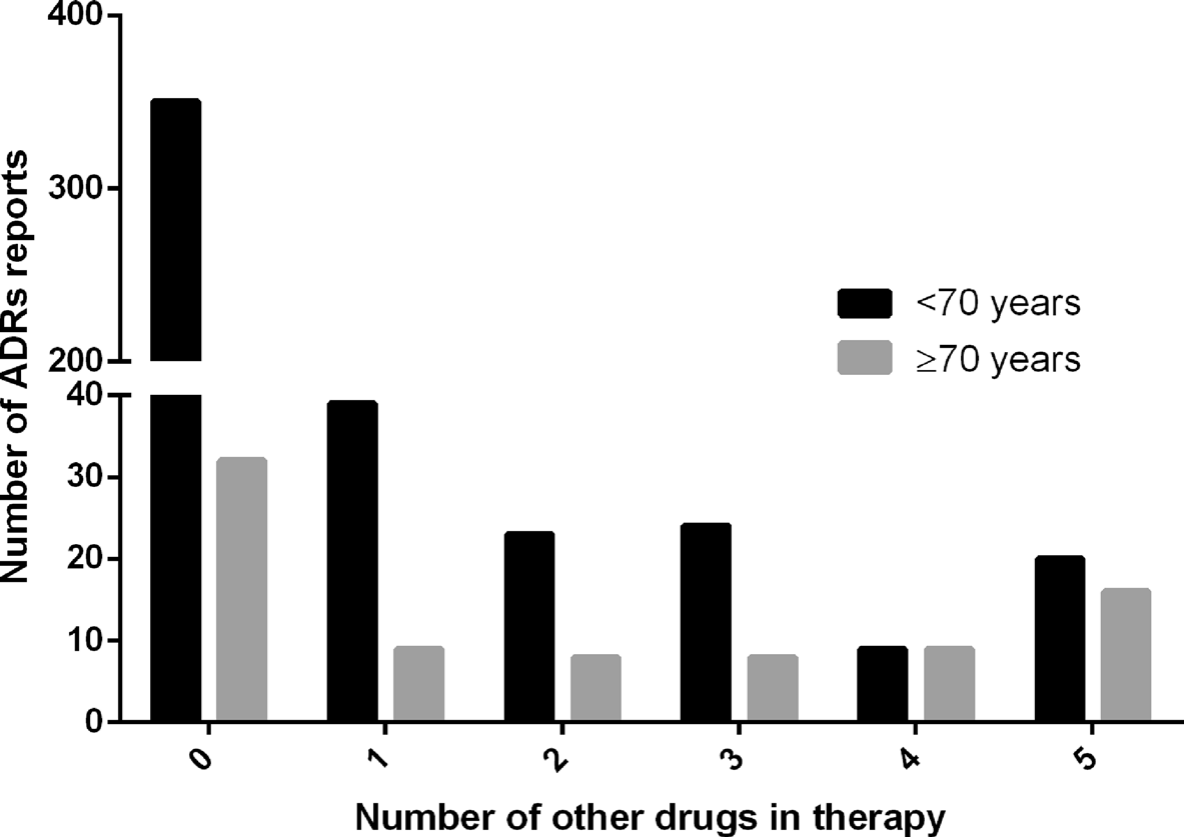
Distribution of ADR reports per number of concomitant drugs

The distribution of ADR reports per number of concomitant drugs is presented in Fig. 2. As concomitant drugs were most frequently reported in consumers over the age of 70 (*p* = 0.047), this age group was separated from the other age groups. Furthermore, common patterns in suspected causal drugs and concomitant drugs were explored. This analysis revealed that diuretics were used concomitantly in 9 out of 20 cases where diosmin/hesperidin was the suspected OTC drug. However, this finding requires further investigation to confirm the possibility of drug-drug interaction.

## Discussion

The results of this study show that the number of ADR reports of OTC drugs has increased through the years, as well as the number of ADR reports in general. It has been recognized that the number of ICSRs in Croatia is high and constantly growing [18]. In this study, ADRs were most frequently reported in women. This was already observed in previously published data from Croatia, but also in other countries [17–19]. Previous studies also reported that women are more likely to use OTC drugs than men [20].

Notably, ADRs included in this study that could have been prevented were accidental exposures among children. A large amount of those reports reflect the need for better patient education about securing children from unsupervised drug ingestion, i.e., childproofing mechanisms. Similar findings were previously observed in the USA and by the Uppsala Monitoring Centre [21, 22]. Most of the ADRs were categorized as non-serious. However, it should be acknowledged that even non-serious ADRs could have a major influence on patients’ quality of life [23].

According to previously published data on ICSRs, physicians were the most common reporters of ADRs [10, 17, 18]. Our finding that pharmacists reported most ADRs of OTC drugs was expected since community pharmacists are involved in patients’ self-medication process. Furthermore, a pharmacist’s intervention, for instance, as a follow up program or identification of OTC drug misuse and abuse, can lead to improved patient outcomes [24, 25]. Several studies concluded that the role of pharmacists in educating consumer of appropriate use of OTC drugs is essential for appropriate use [26–29]. However, the need for improvement of OTC consultation in the pharmacy setting has been recognized [29–31].

Our results reflect the need for raising awareness amongst other health care professionals to be more involved in the reporting of ADRs of OTC drugs and ADRs in general. Previous studies recognized an important role of nurses in detecting and reporting ADRs [32, 33]. It would be reasonable to offer educational programs specifically to nurses or dentists to increase their awareness of ADRs reporting. In order to improve health care professionals’ awareness of the importance of pharmacovigilance and encourage them to report ADRs, several educational interventions have been proposed. At the student level, the majority of interventions were lectures [34–36]. However, in the study by Schutte et al. the students had an opportunity to assess real ADR reports and this kind of education offered students a valuable pharmacovigilance experience, which resulted in increased awareness of ADR reporting [37]. At the health care professional level, education interventions generally aimed to improve their knowledge, which consequently should increase their reporting practice [38, 39]. In the study by Opadeyi et al., awareness of ADR reporting in professionals was additionally reinforced via yearlong monthly text messages resulting in increased reporting practices [40]. Unfortunately, the long term effects of most education interventions remain unknown. The need for the introduction of pharmacovigilance education at the university level has been recognized and in June 2018 a WHO pharmacovigilance core curriculum has been proposed. Key concepts for university teaching were understanding the importance of pharmacovigilance, along with recognizing, preventing, managing and reporting ADRs [41].

In 2017, most of the reports were obtained from consumers. This interesting finding could have been a result of HALMED’s pharmacovigilance campaigns for consumers or the introduction of a mobile application for ADR reporting as a part of the Web-Recognising Adverse Drug Reactions (WEB-RADR) project [18]. Croatia was among first three countries in the European Union that offered this way of spontaneous reporting which enabled patients to easily report ADRs. The study by Oosterhuis et al., conducted on data from 2016, showed that higher proportions of reports through the mobile application compared to other ways of reporting were submitted by consumers in Croatia and the UK [42]. However, the exact route of reports included in this research was not studied and future studies should investigate the impact of the WEB-RADR mobile application on consumers’ reporting.

In the present study, the age group most susceptible to ADRs of OTC drugs were patients aged above 70 years of age. One of the reasons why older adults are in need of improvement in their knowledge of OTC drug safety could be their misuse of OTC drugs [43, 44]. Furthermore, polypharmacy observed especially in this subgroup could also be a cause of ADRs. In the study by Gazibara et al., 10.4% of the included patients aged above 65 years have used ≥5 prescribed drugs, and multiple conditions in patients correlated with multiple OTC drugs used [45]. Wawruch et al. described that elderly patients who considered that the use of several drugs could increase the risk of ADRs were less probable to consider OTC drugs as safe [46]. Irresponsible use of OTC drugs in this age group was previously observed and the limitation of OTC drug availability to pharmacies only was proposed [47]. Use of OTC analgesics, which were excluded from this study, has been associated with increased ADR risk and our results reflect the need to use all OTC drugs with caution in the elderly population [48].

Most of the ADR reports were from consumers who had used only one drug in therapy, the OTC drug, and suspected that it had caused the problem. This finding is important because many previously published investigations outlined the importance of drug interactions and polypharmacy. Moreover, in order to avoid the use of unnecessary drugs or prevent drug related problems, pharmacists should be given access to patients’ charts that would ideally include data on OTC drugs, as well as prescription drugs [3]. It should be noted that in Croatia most OTC drugs are offered for sale only in pharmacies and only a number of OTC drugs are available in specialized stores under pharmacists’ supervision [49]. In some countries, a number of OTC drugs are available outside the pharmacy setting [50]. It is possible that pharmacists’ involvement in the selection of appropriate OTC drugs results in a decreased number of ADRs caused by a drug interaction.

This study has several limitations. Firstly, there is underreporting of ADR data by health care professionals, which is an established limitation in the use of spontaneously reported data. Secondly, there is a possibility that reporters omitted reports about concomitant drugs in patients’ therapy. Moreover, the results of this study may have greater value if interpreted relative to OTC drug consumption in Croatia. However, this data is not available and therefore not included in this study. Furthermore, it is likely that during the 10-year period some of the included OTC drugs may have experienced shortages, and their unavailability on the market could have influenced the number of ADR reports.

## Conclusions

Our study showed an increase in the reporting ADRs of OTC drugs. Specifically, we observed that ADRs were most frequently reported in the elderly and a number of ADRs in pediatric patients were through accidental product exposure. Health care professionals should address concerns in these most vulnerable age groups. Pharmacists most frequently reported ADRs of OTC drugs and consumers’ reporting of ADRs has risen. Other health care professionals (e.g., nurses and dentists) should be encouraged to report ADRs of OTC drugs and proper education must be offered to increase the reporting of ADRs.

## Supplementary information

**Additional file 1.** OTC drugs with reported ADR to HALMED during 10-year period (DOCX 14 kb)

## Data Availability

The data that support the findings of this study are available from HALMED, but restrictions apply to the availability of these data, which were used under license for the current study, and so are not publicly available. Data are however available from the authors upon reasonable request and with permission of HALMED.

## Abbreviations

ADR: Adverse drug reaction
ATC: Anatomical Therapeutic Chemical
HALMED: Agency for Medicinal Products and Medical Devices of Croatia
ICSRs: Individual case safety reports
MedDRA: Medical dictionary for Regulatory Activities
OTC: Over-the-counter
WEB-RADR: Web-Recognising Adverse Drug Reactions
WHO: World Health Organization
