# Supplementary material for: Analysis of spontaneous reporting of suspected adverse drug reactions for non-analgesic over-the-counter drugs from 2008 to 2017
Source: BMC Pharmacol Toxicol. 2019 Oct 18;20:60. doi: 10.1186/s40360-019-0338-2 (PMC6798506; doi:10.1186/s40360-019-0338-2)
Supplement: Supplementary file 1 — Additional file 1. OTC drugs with reported ADR to HALMED during 10-year period (DOCX 14 kb) [file 40360_2019_338_MOESM1_ESM.docx]

OTC drugs with reported ADR to HALMED during 10-year period

| OTC drug generic name | Number of ADRs reported (%) |
| --- | --- |
| diosmin/hesperidin | 42 (7.7) |
| chlorhexidine/lidocaine | 34 (6.2) |
| bromhexine | 31 (5.7) |
| ulipristal | 28 (5.1) |
| acetylcysteine | 27 (4.9) |
| clotrimazole | 24 (4.4) |
| ambroxol | 20 (3.7) |
| *Hedera helix*, folium | 20 (3.7) |
| *Serenoa repens*, fructus | 20 (3.7) |
| aciclovir | 19 (3.5) |
| ranitidine | 19 (3.5) |
| naftifine | 15 (2.7) |
| dimetindene | 13 (2.4) |
| oxymetazoline | 13 (2.4) |
| *Ginkgo biloba*, folium | 12 (2.2) |
| bisacodyl | 10 (1.8) |
| simethicone | 10 (1.8) |
| racecadotril | 10 (1.8) |
| povidone-iodine | 9 (1.6) |
| levonorgestrel | 9 (1.6) |
| vitamins A, B1, B2, B3, B5, B6, B9, B12, C, D, E and biotin, minerals Fe, Ca, Cu, Mg, Mn, P and Zn | 9 (1.6) |
| xylometazoline | 9 (1.6) |
| loratadine | 8 (1.5) |
| chloropyramine | 8 (1.5) |
| xylometazoline*/*dexpanthenol | 7 (1.3) |
| heparin sodium | 7 (1.3) |
| hydrotalcite | 7 (1.3) |
| dimenhydrinate | 6 (1.1) |
| fexofenadine | 5 (0.9) |
| *Valeriana officinalis*, radix | 5 (0.9) |
| vitamins B1, B2, B3, B5, B6 and B12 | 5 (0.9) |
| nicotine | 5 (0.9) |
| butamirate | 5 (0.9) |
| *Allium cepa*, bulbus | 4 (0.7) |
| loratadine/pseudoephedrine | 4 (0.7) |
| ketoconazole | 4 (0.7) |
| benzydamine | 4 (0.7) |
| *Silybum marianum*, fructus | 4 (0.7) |
| hyoscine butylbromide | 3 (0.5) |
| minoxidil | 3 (0.5) |
| aluminium hydroxide/magnesium carbonate | 3 (0.5) |
| hypromellose | 3 (0.5) |
| guaifenesin | 3 (0.5) |
| lactulose | 3 (0.5) |
| tyrothricin | 3 (0.5) |
| benzydamine/cetylpyridinium chloride | 3 (0.5) |
| 2,4 - dichlorobenzyl alcohol/amylmetacresol | 3 (0.5) |
| bifonazole | 3 (0.5) |
| octenidine/phenoxyethanol | 3 (0.5) |
| *Hypericum perforatum*, herba | 2 (0.4) |
| pantoprazole | 2 (0.4) |
| hexetidine | 2 (0.4) |
| *Bifidobacterium animalis* | 2 (0.4) |
| naphazoline | 2 (0.4) |
| *Angelica archangelica*, radix | 2 (0.4) |
| vitamin C | 2 (0.4) |
| activated charcoal | 1 (0.2) |
| *Arctostaphylos uva-ursi*, folium | 1 (0.2) |
| fenticonazole | 1 (0.2) |
| vitamin D and mineral Ca | 1 (0.2) |
| cetirizine | 1 (0.2) |
| calcium carbonate/magnesium carbonate | 1 (0.2) |
| *Eucalyptus globulus*, aetheroleum | 1 (0.2) |
| lactic acid | 1 (0.2) |
| *Cimicifuga racemosa*, rhizoma | 1 (0.2) |
|  |  |
